# Down-regulation of interleukin 7 receptor (IL-7R) contributes to central nervous system demyelination

**DOI:** 10.18632/oncotarget.16081

**Published:** 2017-03-10

**Authors:** Xudan Lei, Shijiao Cai, Yang Chen, Jianlin Cui, Yajie Wang, Zongjin Li, Yuhao Li

**Affiliations:** ^1^ Key Laboratory of Tumor Microenvironment and Neurovascular Regulation, Nankai University School of Medicine, Tianjin 300071, China

**Keywords:** interleukin 7 receptor (IL-7R), demyelination, myelin basic protein, myelination, zebrafish

## Abstract

Interleukin 7 receptor (IL-7R) has been associated with the pathogenesis of multiple sclerosis (MS), though the mechanisms are not clear. Because myelin expression is highly conserved between zebrafish and mammals, zebrafish have become an ideal model for studying demyelination. We used a transgenic (Tg; *mbp:nfsB-egfp*) zebrafish line in which oligodendrocytes expressed green fluorescent protein (GFP) from the larval stage to adulthood. Exposing adult transgenic zebrafish to metronidazole induced demyelination that resembled the morphological changes associated with the early stages of MS. The metronidazole-induced demyelination was confirmed by magnetic resonance imaging (MRI) for the first time. Microarray analysis revealed down-regulation of IL-7R during demyelination. Targeted knockdown of IL-7R demonstrated that IL-7R is essential for myelination in embryonic and larval zebrafish. Moreover, IL-7R down-regulation induced signaling via the JAK/STAT pathway leading to apoptosis in oligodendrocytes. These findings contribute to our understanding of the role of IL-7R in demyelination, and provide a rationale for the development of IL-7R-based therapies for MS and other demyelinating diseases.

## INTRODUCTION

Demyelinating diseases are characterized by lesions that are associated with loss of myelin, while sparing axons in the nervous system [1]. Demyelination can affect the central nervous system (CNS), and is associated with the pathophysiology of various neurodegenerative diseases including demyelinating disorders like multiple sclerosis (MS) [1]. Although the specific mechanisms of demyelination remain unclear, they include autoimmune and inflammatory responses involving T- and B-lymphocytes [2]. Several pro- and anti-inflammatory cytokines affect the demyelination, both in MS patients and in an experimental autoimmune encephalomyelitis (EAE) animal model [3].

Interleukin 7 (IL-7) is a vital cytokine necessary for the development and survival of T- and B-lymphocytes [4–7]. IL-7 mediates its actions through binding to its specific receptor, IL-7 receptor (IL-7R) [8]. IL-7R is a heterodimer that consists of IL-7R alpha subunit (IL-7Rα) and the common gamma chain (γc), which is shared by the cytokine receptors for IL-2, IL-4, IL-9, IL-15, and IL-21 [9, 10]. The IL-7/IL-7R pathway affects extracellular matrix remodeling and development, and homeostasis of B and T cells [11]. Recent studies have indicated that the IL-7R may be associated with the pathogenesis of MS [12, 13]. Single nucleotide polymorphisms in the gene encoding IL-7Rα have been detected in MS patients with different ethnic backgrounds [14–17]. In the blood of MS patients who have the *IL-7R*α mutation, expression of soluble IL-7Rα was increased, whereas expression of the IL-7Rα dimer on the membrane surface was decreased [18]. In the EAE animal model, the IL-7Rα expression was observed on astrocytes and oligodendrocytes; mice lacking IL-7Rα on hematopoietic cells develop severe EAE [3]. In addition, the IL-7/IL-7R signaling has been implicated in the regulation of Th17 and Th1 cell dynamics in EAE [19]. These findings highlight the importance of investigating the role of IL-7R in demyelination.

The pathogenesis of demyelination was initially investigated using rodents’ animal models. Besides the EAE model, chemical methods, such as feeding cuprizone or injecting ethidium bromide into the central nervous system, have been used to induce demyelination in rodents [20]. Since the myelin structure, myelination, and expression of myelin-related genes are highly conserved between zebrafish and mammals, zebrafish has become an ideal model organism to study demyelination in recent years [21–23]. The transgenic (Tg) fish Tg (*plp-egfp*), Tg (*olig2-egfp*), Tg (*sox10-egfp*), and Tg (*mbp-egfp*) derived GFP by different gene promoters were generated [24–27]. However, demyelination cannot be specifically induced in these transgenic lines. We have recently generated a Tg (*mbp:nfsB-egfp*) transgenic line using nitroreductase/metronidazole cell ablation technology [28]. The *myelin basic protein* gene (*mbp*), which is specifically expressed in oligodendrocyte lineage cells, was used as the marker gene to observe the myelin sheath. In the Tg (*mbp:nfsB-egfp*) line, the oligodendrocytes specifically and continuously express a fusion protein composed of enhanced green fluorescent protein (EGFP) and nitroreductase from the *mbp* promoter [28]. The nitroreductase-metronidazole system allows a targeted cell ablation because the nitroreductase enzyme can convert metronidazole into a cytotoxic agent that only induces death of nitroreductase-positive cells [29]. Therefore, the Tg (*mbp:nfsB-egfp*) zebrafish is a heritable and stable transgenic line that provides a powerful tool to explore the mechanisms governing demyelination.

In this study, we used adult, larval, and embryonic zebrafish models to investigate the mechanisms regulating demyelination. Microarray analysis of gene expression profiles revealed a downregulation of IL-7R during demyelination. Finally, we analyzed the effect of IL-7R suppression on myelination, and investigated the responsible mechanisms.

## RESULTS

### Demyelination in Tg (*mbp:nfsB-egfp*) adult zebrafish induces inflammatory response in the spinal cord

**Figure 1:**
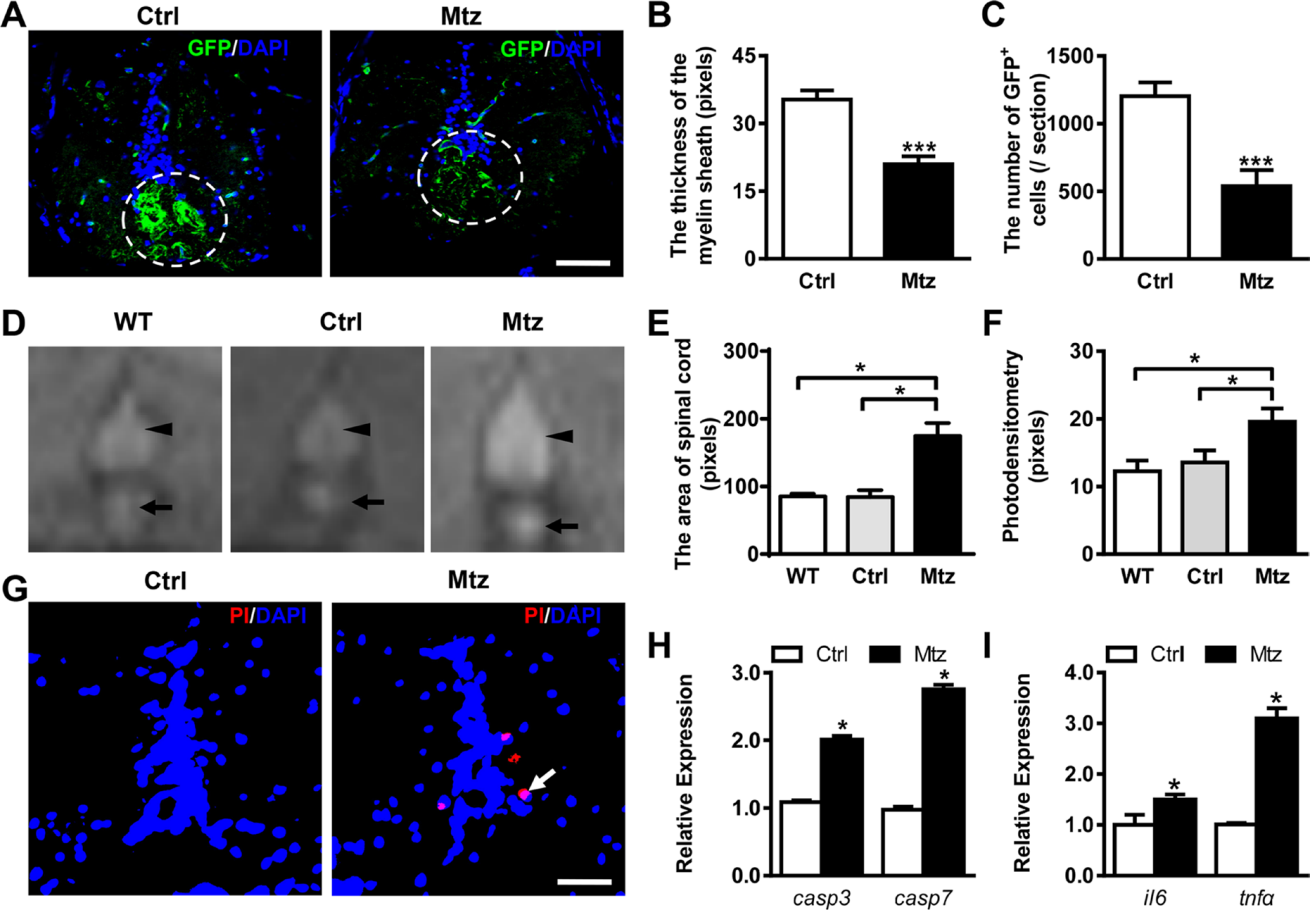
Metronidazole treatment leads to effective demyelination in adult Tg (*mbp:nfsB-egfp*) zebrafish (**A**) Immunostaining of GFP expression on transverse sections taken through the spinal cords of the control Tg (*mbp:nfsB-egfp*) (Ctrl, left view) and the metronidazole treated Tg (*mbp:nfsB-egfp*) (Mtz, right view). Note that there were fewer GFP-positive cells that failed to form the concentric rings structure in the spinal cord in Mtz group. (**B** and **C**) The quantitative comparison of the thickness of the myelin sheath (B) and the number of GFP positive cells (C) between the control (*n* = 8) and metronidazole treated adults (*n* = 9). There were statistically significant differences between the two groups (Student's *t-test*, ***P < 0.001). (**D**) The images of spinal cords (arrowheads) and notochord remnants (arrows) taken using MRI scanning from wild-type (WT), control Tg (*mbp:nfsB-egfp*) adult (Ctrl) and metronidazole treated Tg (*mbp:nfsB-egfp*) adult (Mtz). (**E** and **F**) The statistical analysis of the area and photodensity of the spinal cords from three groups (*n* = 3 in each group). Note that the area and photodensity in Mtz group are significantly increased compared to the WT and Ctrl groups (ANOVA, *P < 0.05). (**G**) The TUNEL staining on transverse section taken through the spinal cords of control Tg (*mbp:nfsB-egfp*) adult (Ctrl, left view) and metronidazole treated Tg (*mbp:nfsB-egfp*) (Mtz, right view). (**H**) The relative expression of *capspase3* (*casp3*) and *caspase7* (*casp7*) mRNA in Ctrl and Mtz groups. (**I**) The relative expression of *interleukin 6 (il6)* and *tumor necrosis factor alpha* (*tnfα*) mRNA in Ctrl and Mtz groups. Note that the expression of *casp3*, *casp7*, *il6* and *tnfα* in the Mtz group is significantly increased compared to the Ctrl group (*n* = 3 in each group; Student's *t* test, *P < 0.05). Dorsal is up in A, D and G. Scale bar: A, 50 μm; G, 100 μm.

To evaluate the efficiency of cell depletion in adult zebrafish, 4-month-old Tg (*mbp:nfsB-egfp*) zebrafish were exposed to 5 mM metronidazole for 72 h, followed by immunohistochemistry (IHC) of transverse sections of the spinal cord. In control adult zebrafish, the GFP-expressing cells were detected mainly in the ventral spinal cord and the myelin sheath formed in concentric rings around the axons. However, after metronidazole treatment, the number of GFP-expressing cells in the ventral spinal cord was remarkably decreased, and the myelin sheath was incomplete and difficult to identify (Figure 1A). To quantify these findings, the thickness of the myelin sheath and the total area occupied by the GFP-positive cells were scored for both control and metronidazole treated groups. The myelin sheath of the spinal cords in metronidazole treated fish was significantly thinner than that in the control group (Figure 1B; Student's *t* test, ***P < 0.001). Moreover, the GFP-positive area in the spinal cords from metronidazole treated group was remarkably reduced (Figure 1C; Student's *t* test, ***P < 0.001). Therefore, exposure to 5 mM metronidazole for 72 h led to an effective ablation of oligodendrocytes in the spinal cord of Tg (*mbp:nfsB-egfp*) adult zebrafish.

MRI is a useful technique to detect the pathological changes of the brain or spinal cord and to monitor the progression of MS [30]. The recent advent of ultrahigh field MRI (7T and more) offers an opportunity to improve the identification of neocortical lesions [31]. In the present study, cross-sectional scanning of the spinal cords of the three groups of adult fish was performed using a 7.0T MRI animal instrument (Supplementary Figure 1). The signal intensity of the spinal cords (Figure 1D, arrowheads) and notochord remnants (Figure 1D, arrows) was increased compared to the surrounding skeletal muscles, which made them easy to identify by T_2_-weighted imaging. Compared with wild-type and control adults, the spinal cord of the metronidazole treated group showed an expanded signal area (Figure 1D). We then scored the total area and photodensity of the spinal cords in wild-type, control, and metronidazole treated groups. No significant difference was found between wild-type and control groups. However, the total area and photodensity of the spinal cords in the metronidazole treated group were significantly increased (Figure 1E and 1F; ANOVA, *P < 0.05), indicating the edema of spinal cords. High water diffusion characteristics within the spinal cord may provide important information about the structural nature of spinal cord pathology [32]. To measure apoptosis, we performed TUNEL assay and qRT-PCR. The results showed an increased number of apoptotic cells in the spinal cord following demyelination (Figure 1G, arrow). In addition, expression of two apoptosis-related genes, caspase 3 and caspase 7, increased significantly in the metronidazole treated group (Figure 1H; Student's *t* test, *P < 0.05). We also measured expression of two inflammatory cytokines, interleukin-6 (IL6) and tumor necrosis factor-alpha (TNFα). Both *IL6* and *TNFα* were increased following demyelination (Figure 1I; Student's *t* test, *P < 0.05). Combined with the IHC data, these results indicate that the metronidazole-induced demyelination triggers an inflammatory response in the spinal cord of adult Tg (*mbp:nfsB-egfp*) zebrafish.

### *IL-7R* is down-regulated following demyelination

**Figure 2:**
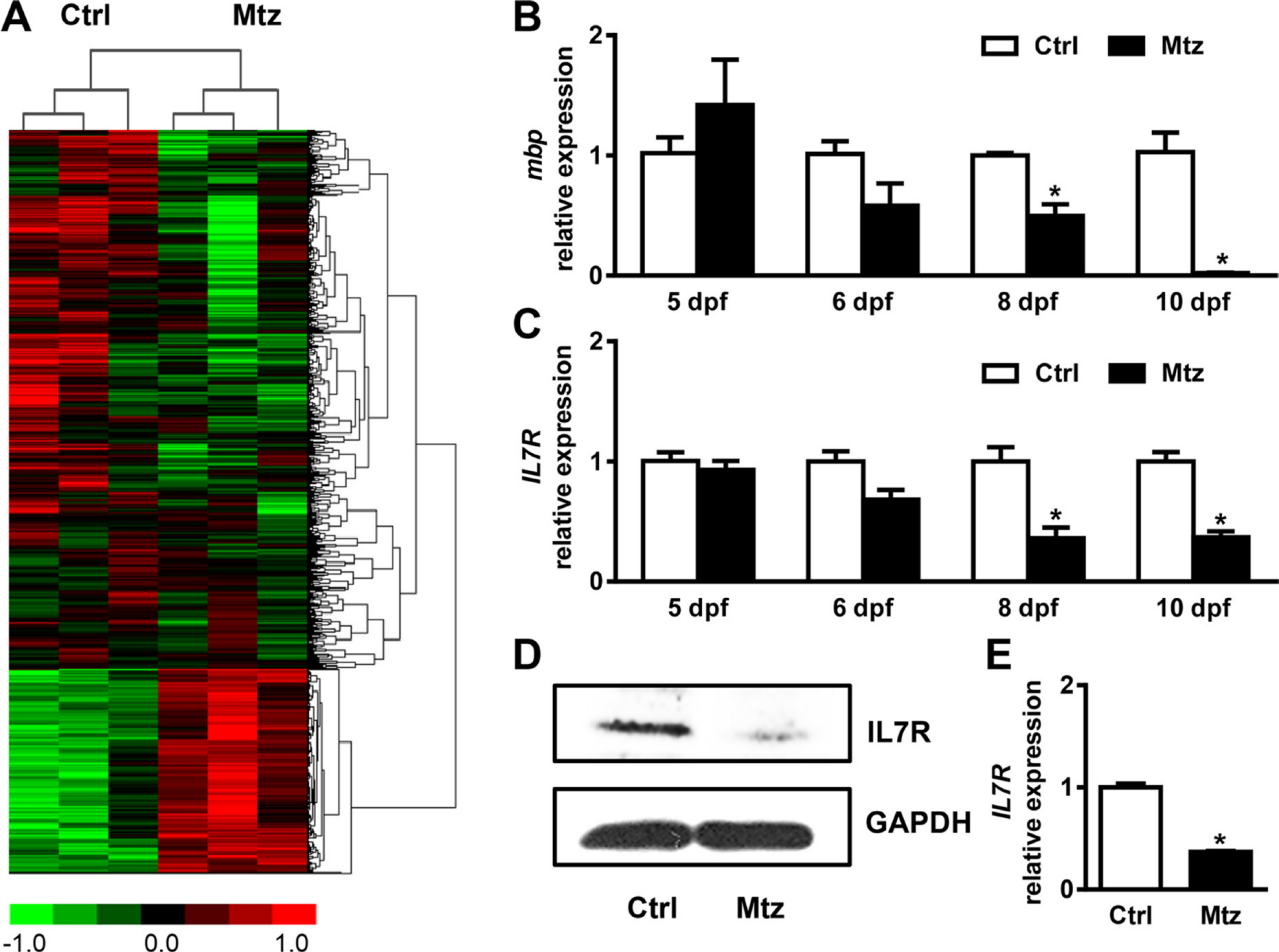
*IL-7R* is down-regulated during targeted demyelination (**A**) The heat map highlighting the differential gene expression profile and cluster analysis following metronidazole treatment assessed by cDNA microarray. (**B**) The relative *mbp* mRNA expression in control Tg (*mbp:nfsB-egfp*) larvae (Ctrl) and metronidazole treated Tg (*mbp:nfsB-egfp*) larvae (Mtz) by qRT-PCR at 5, 6, 8, 10 dpf. Note that the *mbp* expression was decreased at 8 dpf and 10 dpf (Student's *t* test, *P < 0.05). (**C**) The relative *IL-7R* mRNA expression in control Tg (*mbp:nfsB-egfp*) (Ctrl) and metronidazole treated Tg (*mbp:nfsB-egfp*) larvae (Mtz) by real-time PCR at 5, 6, 8 and 10 dpf. Note that the *mbp* mRNA at 8 dpf and 10 dpf shows a decreased expression level (Student's *t* test, *P < 0.05). (**D**) The expression of IL-7R protein by western blotting at 10 dpf. (**E**) The *IL-7R* mRNA relative expression in sorted GFP-positive cells in Ctrl and Mtz groups. Note that the *IL-7R* expression level in the metronidazole group is significantly decreased compared to the Ctrl group (*n* = 3 in each group; Student's *t* test, *P < 0.05).

To identify the genes involved in the demyelination in our zebrafish model, Tg (*mbp:nfsB-egfp*) larvae were continuously exposed to 5 mM metronidazole from day 5 post fertilization (dpf). Then, a microarray analysis was performed using RNA isolated from the control and metronidazole treated larvae to identify the alterations in gene expression at 10 dpf (Gene Expression Omnibus accession GSE89215). The criteria for differential expression were the following: ≥ 2-fold for upregulation and ≤ 0.5-fold for downregulation. 1364 genes showed significant changes in signal intensity, among which 271 genes were upregulated and 1093 genes were downregulated (Figure 2A). Twelve relative gene ontology (GO) cluster changes (Supplementary Figure 2A) and 25 relative pathway changes (Supplementary Figure 2B) were detected. Interestingly, we found that the expression of *IL-7R* in metronidazole-treated larvae was significantly decreased (fold change = 0.44; Supplementary Figure 3). The global signal transduction network also showed an inhibition of *IL-7R* (Supplementary Figure 4A). We analyzed the network and interacting partners with String-db software (http://www.string-db.org/), and found that *IL-7R* was experimentally determined to interact with JAK1 and JAK3 (Supplementary Figure 4B).

To investigate the relationship between *mbp* and *IL-7R*, first, the larvae were collected at 5, 6, 8, and 10 dpf, and the relative expression of *mbp* mRNA was quantified compared with the control larvae at each time point. The expression of *mbp* mRNA was decreased in a time-dependent manner (Figure 2B). Before metronidazole treatment, the expression of *mbp* mRNA in larvae from the metronidazole treated group was slightly higher than in the control group. After 1 day post treatment (at 6 dpf), the expression of *mbp* mRNA decreased, although no statistical difference was found between the two groups. However, at 8 and 10 dpf, the expression of *mbp* mRNA in larvae from the metronidazole treated group was remarkably decreased compared to the control group (Figure 2B; Student's *t* test, *P < 0.05). Next, qRT-PCR and western blotting were performed to validate the down-regulation of *IL-7R*. The treated larvae of Tg (*mbp:nfsB-egfp*) were collected at 5, 6, 8, and 10 dpf, and the expression of *IL-7R* mRNA was analyzed (Figure 2C). The *IL-7R* mRNA expression in treated larvae was dramatically lower than in control larvae at 8 and 10 dpf (Figure 2C; Student's *t* test, *P < 0.05). The IL-7R protein level in the metronidazole group was also lower than in the control group at 10 dpf (Figure 2D).

To check the status of *IL-7R* expression specifically in oligodendrocytes, the same number of GFP-positive cells were sorted and collected from the control group and the metronidazole treated group. Cross-intron primers were designed to detect whether the oligodendrocytes expressed *IL-7R*. The relative expression of *IL-7R* in oligodendrocytes from the metronidazole treated group was significantly decreased compared to the control group (Figure 2E; Student's *t* test, *P < 0.05). Therefore, IL-7R down-regulation occurs not only in the whole body but also in oligodendrocytes.

### Knockdown of *IL-7R* delays myelination in the spinal cord

**Figure 3:**
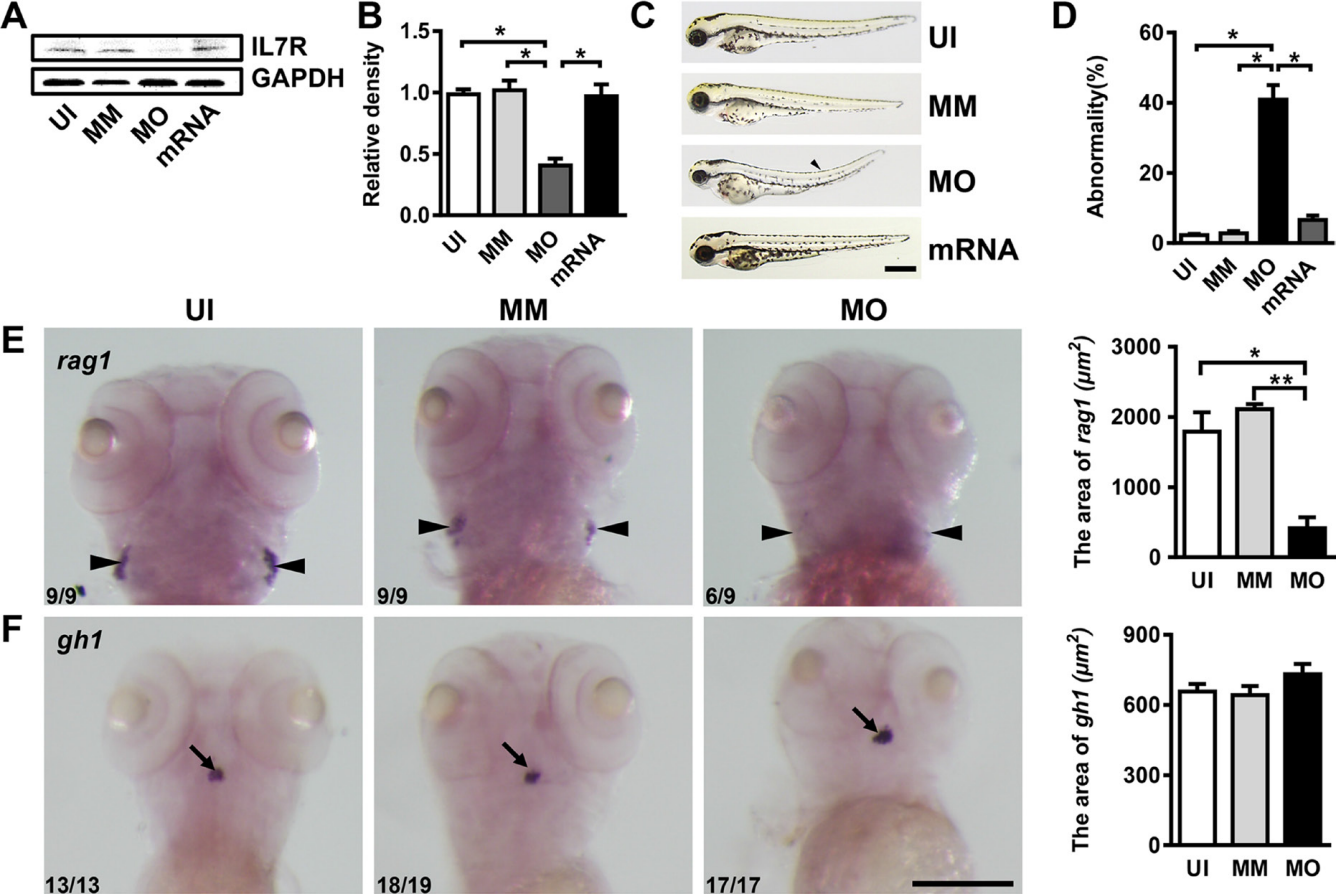
*IL-7R*-targeted morpholino oligonucleotides cause abnormal phenotypes and affect thymus development (**A**) The expression of IL-7R protein of uninjected (UI), mismatch control (MM), *IL-7R*-morphant (MO) and *IL-7R*-rescue (mRNA) groups at 72 hpf. (**B**) Note that the IL-7R protein expression was significantly suppressed in *IL-7R*-morphants (ANOVA, *P < 0.05). (**C**) The gross development of UI, MM, MO and *IL-7R*-rescue (mRNA) embryos at 72 hpf. Some of the *IL-7R*-morphants show a slight curve in body axis (arrowhead). (**D**) The rate of abnormal phenotype increases in MO group (ANOVA, *P < 0.05). (**E** and **F**) The images of whole mount *in-situ* hybridization with mRNA probes *recombination activating gene 1* (*rag1*) and *growth hormone1* (*gh1*) in UI, MM and MO at 5 dpf. E. Note that the expression of *rag1* is significantly decreased in *IL-7R* morphants (E; ANOVA, *P < 0.05, **P < 0.01) while the *gh1* positive signals are similar among all three groups (F). Scale bar: C. 500 μm; E. and F., 200 μm.

In this study, an *IL-7R*-targeted morpholino (MO) was used to inhibit the translation of the *IL-7R* gene. At 72 hours post fertilization (hpf), the IL-7R protein expression was specifically reduced (Figure 3A and 3B; ANOVA, *P < 0.05). The gross development of some *IL-7R* morphants showed a slight curve in the body axis at 72 hpf (Figure 3C, arrowhead). The abnormality rate in *IL-7R* morphants was significantly increased compared to un-injected and mismatch controls at 72 hpf (Figure 3D; ANOVA, *P < 0.05). To prove the specificity of *IL-7R*-targeted knockdown, we performed the rescue experiment. We co-injected the *IL-7R* MO with *IL-7R* mRNA into embryos and quantified the expression of IL-7R protein by western blotting. Following *IL-7R* mRNA rescue, the IL-7R protein was significantly increased (Figure 3A–3B; ANOVA, *P < 0.05). Moreover, the *IL-7R* mRNA rescue embryos showed a similar gross development compared to the un-injected and mismatch embryos (Figure 3C). In zebrafish, *IL-7R* deficiency leads to the absence of T cells, affecting only the differentiation of lymphocytes in the thymus, while the hypophysis develops normally [33]. Therefore, the targeted knockdown of *IL-7R* was further verified by whole-mount *in situ* hybridization with probes for *rag1* and *gh1*, which label the thymus and hypophysis at 5 dpf, respectively. The *rag1*-expressing cells were evident in the bilateral thymus anlage of un-injected and mismatch control larvae. The cellular localization of *rag1* signals in *IL-7R* morphants was similar in un-injected and mismatch animals; however, the area of *rag1*-expressing cells was reduced dramatically in *IL-7R* morphants (Figure 3E; ANOVA, *P < 0.05). In contrast, the *gh1* signals were detected in the hypophysis of larvae from the three groups, and no significant differences were found (Figure 3F). These results indicate that an injection of *IL-7R* morpholino (2 ng) can specifically knockdown *IL-7R* until 5 dpf, thereby providing a model for evaluating the function of IL-7R in myelination.

**Figure 4:**
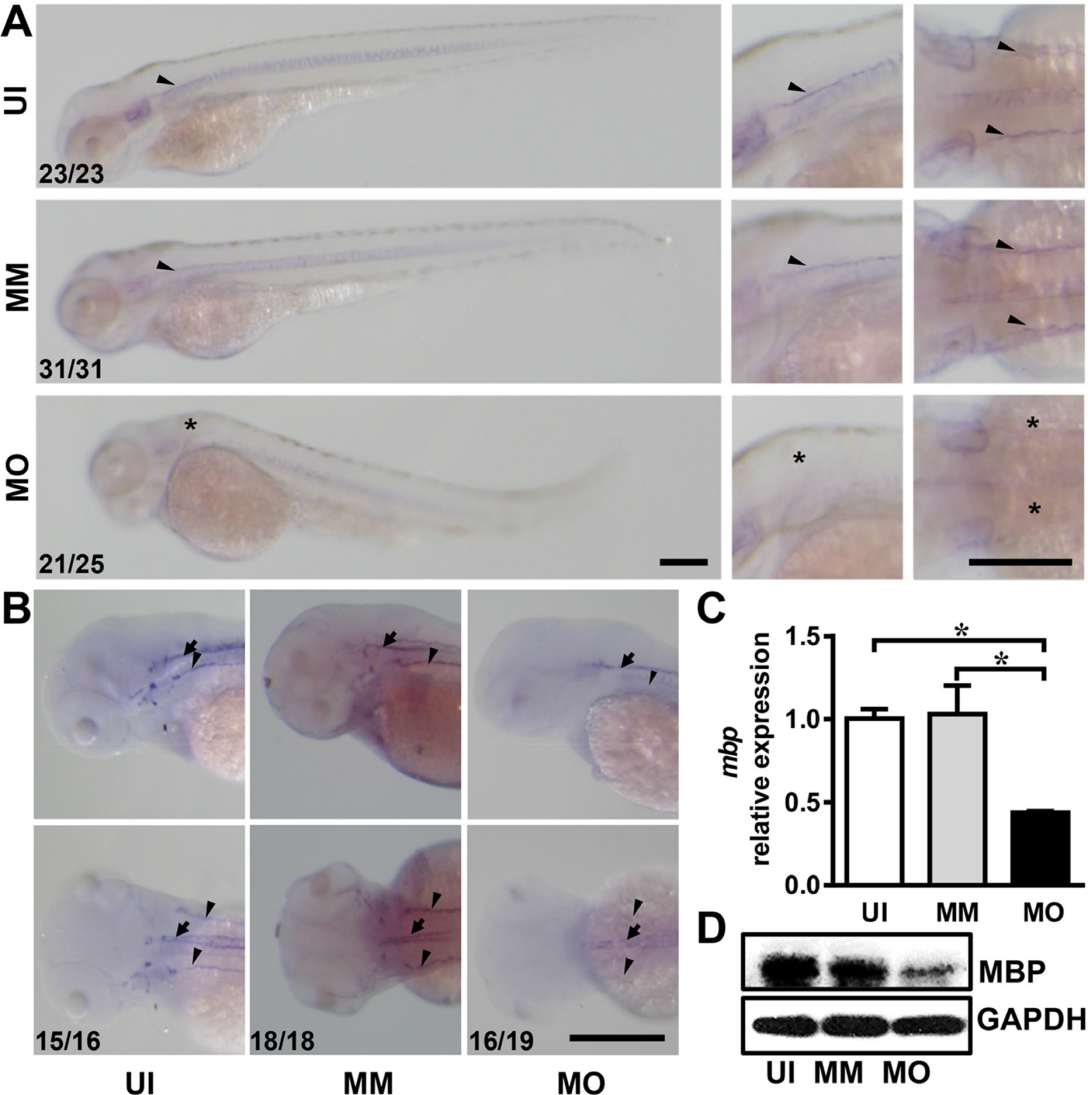
*IL-7R* knockdown delays myelination in the nervous system (**A**) The images of *mbp* mRNA expression in embryos from uninjected (UI), mismatch control (MM) and *IL-7R* morphant (MO) at 3 dpf. The second and third lines are magnified images in lateral view and dorsal view. Note that the *mbp*-expressing cells in *IL-7R* morphant are absent at 3 dpf (asterisks). (**B**) The images of *mbp* expression in the spinal cord (arrows) and lateral line nerves (arrowheads) of the larvae from the UI, MM and MO groups at 4dpf. The *mbp* positive cells distribute along the lateral line nerves and the spinal cord in UI and MM larvae. In MO larva, the *mbp* signals are only detected along the spinal cord. (**C**) The relative expression of *mbp* mRNA expression among three groups using qRT-PCR at 4 dpf (*n* = 3 in each group). Note that the expression level in the MO group is decreased compared to the UI and MM groups (ANOVA, *P < 0.05). (**D**) The expression of MBP protein by western blotting at 4 dpf. Scale bar: A. and B., 200 μm.

*Mbp* was then used as a marker to investigate myelination by whole-mount *in situ* hybridization. At 3 dpf, *mbp* was expressed strongly in Schwann cells linearly along the lateral line of the trunk in un-injected and mismatch controls (Figure 4A, arrowheads). However, the *mbp*-expressing cells were absent in *IL-7R* morphants (Figure 4A, asterisks). At 4 dpf, the *mbp*-expressing cells from un-injected and mismatch groups were distributed in the Schwann cells along the lateral line nerves (Figure 4B, arrowheads) as well as in the oligodendrocytes in the spinal cord (Figure 4B, arrows), which matched the location of myelinated axons at this stage [21]. In *IL-7R* morphants (MO), weak *mbp*-positive signals were detected only along the spinal cord (Figure 4B). The expression of *mbp* mRNA in *IL-7R* morphants at 4 dpf was significantly decreased compared to un-injected and mismatch controls (Figure 4C; ANOVA, *P < 0.05). The MBP protein level was also decreased in *IL-7R* morphants (Figure 4D). These findings suggest that myelination in both the central and peripheral nervous system is delayed following the knockdown of *IL-7R*.

### IL-7R down-regulation reduces expression of *bcl2* through the JAK/STAT signaling pathway

**Figure 5:**
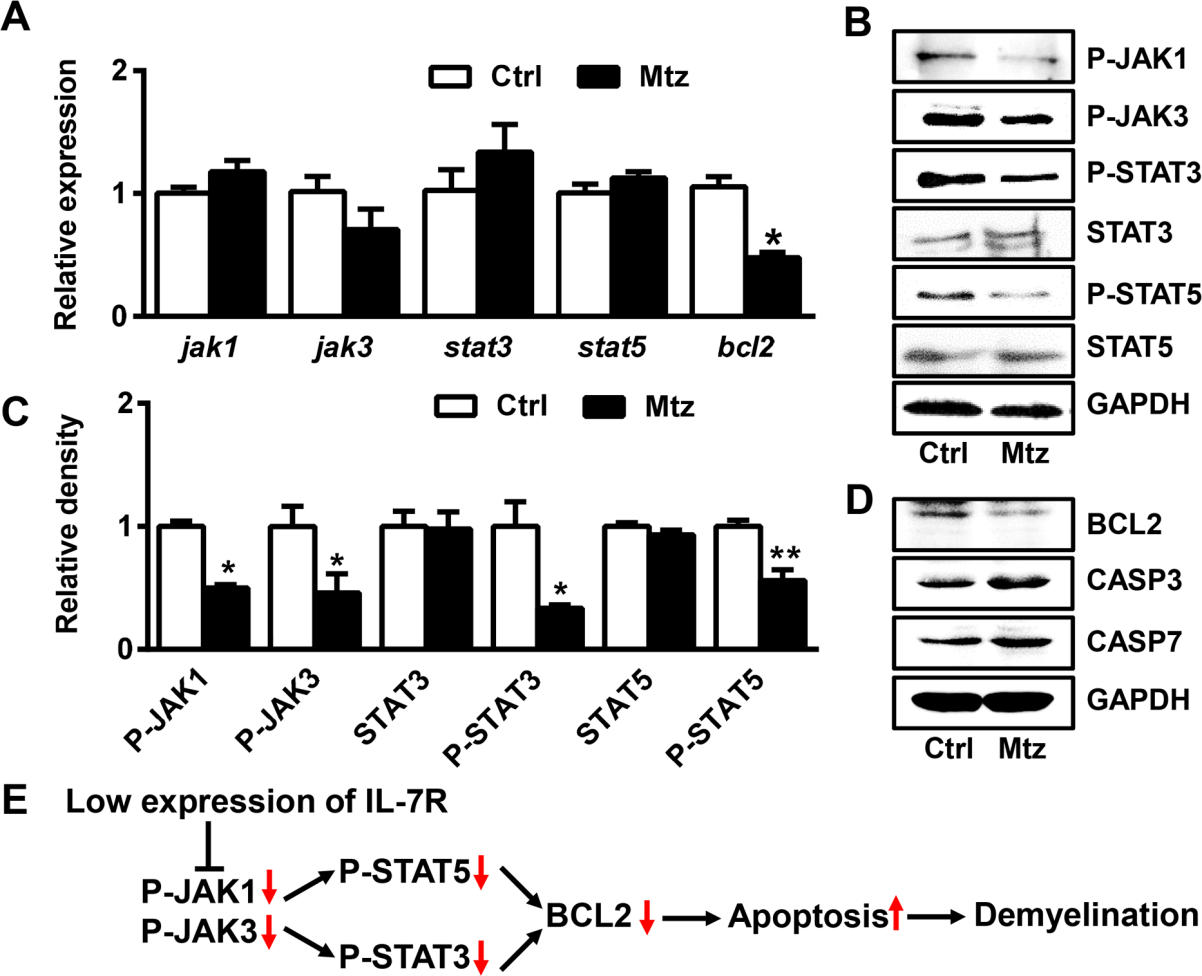
*IL-7R* down-regulation induces apoptosis of oligodendrocytes via the JAK/STAT/BCL2 signaling pathway (**A**) The expression of *jak1*, *jak3*, *stat3*, *stat5* and *bcl2* mRNA measured by qRT-PCR in theTg (*mbp:nfsB-egfp*) (Ctrl) and metronidazole treated (Mtz) groups at 10 dpf. Note that only the expression of *bcl2* in Mtz group was decreased compared to the Ctrl group (*n* = 3; Student's *t* test, *P < 0.05). (**B**) The expression of P-JAK1, P-JAK3, P-STAT3, STAT3, P-STAT5 and STAT5 proteins in Tg (*mbp:nfsB-egfp*) larvae from the Ctrl and Mtz groups at 10 dpf. (**C**) Note that the expression of P-JAK1, P-JAK3, P-STAT3 and P-STAT5 was decreased in larvae from the Mtz group (Student's *t* test, *P < 0.05, **P < 0.01). (**D**) The expression of BCL2, CASPASE3 (CASP3) and CASPASE7 (CASP7) proteins by western blotting in Tg (*mbp:nfsB-egfp*) larvae from the Ctrl and Mtz groups at 10 dpf. (**E**) Putative model outlining the *IL-7R* down-regulation in demylination.

To explore the possible mechanisms governing demyelination, we first examined gene expression of *jak1*, *jak3*, *stat3*, *stat5* and *bcl2* in control and metronidazole treated groups at 10 dpf. No difference was found in the expression of *jak1*, *jak3*, *stat3*, and *stat5* between the two groups (Figure 5A). However, the expression of *bcl2* in the metronidazole treated group was significantly decreased compared to the control group (Figure 5A; Student's *t* test, *P < 0.05). Next, we analyzed the protein levels of the aforementioned genes using western blotting (Figure 5B). The phosphorylated protein levels of JAK1, JAK3, STAT3, and STAT5 were decreased in the metronidazole-treated larvae (Figure 5C; Student's *t* test, *P < 0.05, **P < 0.01). In addition, the protein expression of BCL2 was decreased while the protein levels of CASPASE-3 and CASPASE-7 were increased in the metronidazole-treated larvae (Figure 5D). These data indicate that the IL-7R down-regulation reduces expression of BCL2 and phosphorylation of JAK1, JAK3, STAT3, and STAT5.

## DISCUSSION

The main conclusions of this study are the following: 1) Metronidazole treatment of adult Tg (*mbp:nfsB-egfp*) zebrafish induces effective demyelination, which partially simulates the morphological and radiographic changes consistent with the early stage of MS. 2) IL-7R is an essential molecule of myelination in embryonic and larval zebrafish. 3) IL-7R down-regulation functions via the JAK/STAT signaling pathway and eventually causes an increase in the apoptosis of oligodendrocytes (Figure 5E).

MS is an autoimmune demyelinating disease with an unknown etiology that mainly occurs in young adults [34]. Due to the limitation of accessing immunologically active MS tissue samples, development of animal models is critical for clarifying the underlying immune-pathological mechanisms and for testing novel therapeutic approaches. Several animal models have been developed, but no single one can capture the entire spectrum of heterogeneity of MS [35]. The zebrafish, with its accessible and well-characterized embryological and larval development, provides an ideal platform for studying the pathogenesis of MS [21]. Magnetic resonance imaging (MRI) has become an important element in the diagnosis of MS [36]. In this study, we verified the MS model of demyelination in Tg (*mbp:nfsB-egfp*) zebrafish by 7.0T-MRI. T_2_-weighted images revealed abnormally high signals detected in the spinal cord and notochord remnant, which were displayed as an expansion of the signal area and an increase of photodensity in demyelinated zebrafish. B cells, plasma cells, and follicular dendritic cells are activated in about 40% of patients with progressive MS [37]. Some patients also have an acute swelling in the spinal cord at the onset of MS [38]. Our results indicate that the demyelination induced by metronidazole treatment in adult Tg (*mbp:nfsB-egfp*) zebrafish can induce apoptosis of oligodendrocytes and trigger an inflammatory response in the spinal cord. These events partially mimic the early pathological and imaging changes in MS. This is the first study using MRI to reveal the imaging changes of demyelination in zebrafish model of MS. Moreover, the size of the myelin sheath and the integrity of tissue structure were useful to make an objective evaluation of the injuries of the myelin sheath and the pathological changes of the spinal cord in MS [39].

MS is a very heterogeneous disease of the central nervous system (CNS) and several mechanisms have been proposed to explain the underlying pathogenesis [1]. Experimental and clinical studies have indicated that the dysfunction of immune cells helps prevent autoimmune demyelination. For example, the immune-deficient *IL-7Rα*^−/−^ mice are resistant to EAE induced by myelin oligodendrocyte glycoprotein (MOGp35-55) [40]. In addition, human immunodeficiency virus 1 (HIV-1) infection has been associated with a reduced MS risk [41]. However, our results indicate that the expression of *IL-7R* is decreased during demyelination. This discrepancy might be explained by the different animal models used. In our zebrafish model, metronidazole was used to induce the death of oligodendrocytes in a short period. We believe that this method has a little effect on the immune system. In clinical practice, a severe immune inhibition is not very common in patients. On the contrary, the immune system of patients with autoimmune diseases is sometimes in an active state, such as an increase in Th1, CD4^+^, and CD25^+^ T cells [42]. Increased numbers and activation of microglia/macrophages have been also reported [43]. Our results demonstrated decreased expression of *mbp* and *IL-7R* in metronidazole-treated zebrafish. This is consistent with a study demonstrating that the expression of IL-7R is decreased in patients with primary progressive MS [44].

To clarify the role of *IL-7R* in myelination, *IL-7R* was knocked-down in the embryonic zebrafish. Following *IL-7R* knockdown, some morphants showed a shortened body-axis and curving. We speculate that the interference with the development of T cells may affect gross development. No *mbp*-expressing cells were detected at 3 dpf in *IL-7R* morphants. Only weak signals were found along the spinal cord until 4 dpf, and the *mbp* expression in *IL-7R* morphants at 4 dpf was decreased. These findings indicate that although the knockdown of *IL-7R* does not change the positional relationship of the oligodendrocyte lineage cells in the central and peripheral nervous system, it delays their differentiation and maturation.

The α-chain of IL-7R interacts with STAT3 through JAK1, while the γ_c_-chain binds with JAK3 and functions by phosphorylating STAT5 [33, 45]. During metronidazole-induced demyelination, P-JAK1, P-JAK3, P-STAT3, and P-STAT5 levels were significantly reduced. Furthermore, the anti-apoptotic gene, *bcl2*, was decreased, while its two downstream effectors, CASPASE-3 and CASPASE-7 were increased following demyelination. Previous study has revealed that the downregulation of IL-7Rα in neurons and astrocytes could induce apoptosis of neurons in HIV-1-associated dementia [46]. Our results indicate that IL-7R suppression functions via the JAK/STAT signaling pathway, and induces apoptosis, resulting in demyelination. Our findings support the conclusion that the normal expression of IL-7R plays an important and irreplaceable role in maintaining the integrity of structures of the myelin sheath and its function.

In conclusion, zebrafish of the transgenic line Tg (*mbp:nfsB-egfp*) is an ideal animal model for investigating demyelination *in vivo*. Our study provides a new insight into the role of *IL-7R* in demyelination and myelin development. This study will not only contribute to elucidating the comprehensive mechanisms governing demyelination, but will also help develop potential IL-7R-based treatments for demyelinating diseases in humans.

## MATERIALS AND METHODS

### Experimental animals

Wild-type adult fish (AB strain, 4-month old and 3.0–4.0 cm long) were used for Magnetic Resonance Imaging (MRI) scanning. Tg (*mbp:nfsB-egfp*) larvae were used to perform the microarray analysis and characterize the expression of *IL-7R*. Wild-type (AB strain) embryos were used for knockdown experiments. All the fish were raised at 28.5°C with a 10/14-h dark/light cycle [47]. Embryos were collected after natural spawning, housed at 28.5°C, and staged by hours post fertilization (hpf) or days post fertilization (dpf). All protocols for animal procedures were approved by the Institutional Animal Care Committee of Nankai University and conformed to the National Institutes of Health guidelines.

### Metronidazole treatment

Metronidazole (Sigma, M3761, St. Louis, MO, USA) was dissolved in 0.2% dimethyl sulfoxide (DMSO, Sigma V900090) in system water or E3 medium (5 mmol/L NaCl, 0.17 mmol/L KCl, 0.33 mmol/L CaCl_2_, 0.33 mmol/L MgSO_4_, pH 7.2) with vigorous shaking in the dark. For adults, Tg (*mbp:nfsB-egfp*) fish were treated continuously with 5 mM metronidazole-0.2% DMSO-system water for 72 hours in the dark. To control for the possible toxic effects of metronidazole, the same number of wild-type (AB strain) adult fish were also treated with 5 mM metronidazole-0.2% DMSO-system water for 72 h as the wild type group. Meanwhile, the same number of Tg (*mbp:nfsB-egfp*) adults were incubated in 0.2% DMSO-system water alone as the control group. For larvae, Tg (*mbp:nfsB-egfp*) at 5 dpf were treated with 5 mM metronidazole for 5 days in the dark as we described previously [28].

### Immunohistochemistry (IHC)

Adult zebrafish were anesthetized with 0.1% ethyl 3-aminobenzoate methanesulfonate salt (Sigma, A5040) and euthanized immediately. The zebrafish were dissected and fixed in 4% paraformaldehyde (PFA), cryoprotected in 20% sucrose in 0.1M phosphate-buffered saline (PBS, pH 7.4), and frozen in optimal cutting temperature compound (Sakura Finetek, CA, USA). Transverse sectioning (8 μm in thickness) was performed using a cryostat (CM1850, Leica Biosystems Nussloch GmbH, Germany). Immunohistochemistry was performed as described previously [48]. An anti-GFP antibody (1:500; Abcam, ab6556, Cambridge, MA, USA) was used as the primary antibody to label the oligodendrocytes in the spinal cord of Tg (*mbp:nfsB-egfp*).

### TUNEL apoptosis analysis

Cryosections were prepared as described in above. To identify the apoptotic cells, TUNEL assay was performed with TUNEL kit (Promega, G3250; Madison, WI, USA) according to the manufacturer's instructions.

### Magnetic resonance imaging (MRI) acquisition

Adult zebrafish from wild-type, control and metronidazole treated groups were anesthetized with 0.1% MS-222 and euthanized immediately. Then they were quickly positioned laterally in the Biospec 7.0T MRI animal instrument (Bruker, Switzerland) at the National Center for Nanoscience and Technology (Beijing, China). Scanning was performed using the T_2_-TurboRARE sequence along transverse sections with the following parameters: repetition time/echo time = 2900/50 ms, slice thickness = 0.5 mm, flip angle = 90°, matrix = 256 × 256, field of view = 15 × 15 mm, and number of excitations (NEX) = 10.

### Microarray analysis

At 10 dpf, five Tg (*mbp:nfsB-egfp*) larvae from both the metronidazole treated group and the control group were collected, anesthetized, euthanized, and snap-frozen. Total RNA was extracted using an RNeasy Mini Kit (QIAGEN, 74104, Hilden, Germany) and quantified using a nanodrop spectrophotometer (Thermo Scientific, Nano-Drop 1000; Thermo Scientific, DE, USA). The previously described RNA extraction was repeated in three batches of larvae to generate biological replicates of metronidazole treatment and control samples [49]. After quality assessment, 500 ng of RNA from each sample was amplified using the Ambion WT Expression Kit (Carlsbad, CA, USA). Gene expression in the six samples was determined using the GeneChip Zeb Gene 1.0 ST Array (Affymetrix, Santa Clara, CA, USA). The hybridized arrays were scanned using a GeneChip2 Scanner 30007G (Affymetrix).

### Quantitative RT-PCR

Total RNA was isolated from zebrafish larvae using TRIzol reagent (Thermo Scientific), according to the manufacturer's protocol. Total mRNA was reverse-transcribed by the Moloney Murine Leukemia Virus reverse transcriptase (Promega, A5001) using oligo (dT) primers. Quantitative RT-PCR was performed using the SYBR green labeling system (Promega, A6002). The relative expression of mRNA between control and treated groups was normalized to β-actin and evaluated by the 2^−ΔΔCt^ method [50]. The qRT-PCR was performed on three independent batches of control or treated larvae. The gene-specific primer sequences are listed in Table 1.

**Table 1:** Primer sequences for qRT-PCR

Gene	GenBank accession number	Primer Sequences (5′ to 3′)
*il7r*	NM_001113507	Forward: TTACACCAAACATCCCACA
		Reverse: TAGGCTTCTTCTTTACATTCG
*jak1*	NM_131073	Forward: AAACACATCGCCCTGCTCTA
		Reverse: AAAGGGCCGTACTGAACAAA
*stat3*	NM_131479	Forward: GGACTTCCCGGACAGTGAG
		Reverse: ATCGCTTGTGTTGCCAGAG
*jak3*	XM_002663087	Forward: GAGATACCTGCGATTCATCTCT
		Reverse: GTGCTGTAGCAGATGCCCTT
*stat5*	AB018220	Forward: CAGTGCCCCACACCCTT
		Reverse: CATGCCTCGCTACATCCAT
*bcl2*	AY695820	Forward: GTCACTCGTTCAGACCCTCAT
		Reverse: GACGCTTTCCACGCACAT
*caspase3*	NM_131877	Forward: TGTTCTTTATTCAGGCTTGT
		Reverse: CACTGCCATACTTTGTCATC
*caspase7*	NM_001020607	Forward: AGCAATGCCCATCAAGA
		Reverse: ATCGTCAAACTCCGAACCC
*il6*	NM_001261449	Forward: ACGGAAAGATGTCTAACGC
		Reverse: ATAGGGAAGTGCTGGATG
*tnfα*	NM_212859	Forward: TACCGCTGGTGATGGTGT
		Reverse: TGATTGCCCTGGGTCTTA
*actin*	AY222742	Forward: TTCACCACCACAGCCGAAAGA
		Reverse: TACCGCAAGATTCCATACCCA

### Western blotting analysis

At each time point, 30 larvae from control or metronidazole treatment groups were harvested in RIPA lysis buffer containing 1 mM phenylmethanesulfonyl fluoride (PMSF; Sigma, 78830), Protease Inhibitor Cocktail (Promega, G6521), Phosphatase Inhibitor Cocktail 2 (Sigma, P5726), and Phosphatase Inhibitor Cocktail 3 (Sigma, P0044). Western blotting was performed using standard protocols [48]. Eleven primary antibodies were used in this study: anti-IL-7R (1:1500; Abcam, ab180521), anti-MBP (1:500; Anaspec, #55811, Fermont, CA, USA), anti-Phospho-JAK1 (1:1,000; Cell Signaling Technology, #3331, Danvers, MA, USA), anti-Phospho-JAK3 (1:500; Cell Signaling Technology, #5031), anti-STAT3 (1:500; Santa Cruz Biotechnology, sc-7179, TX, USA), anti-Phospho-STAT3 (1:200; Cell Signaling Technology, #9145), anti-STAT5 (1:500; Santa Cruz Biotechnology, sc-836), anti-Phospho-STAT5 (1:200; Cell Signaling Technology, #9351), anti-BCL2 (1:1000; BD Biosciences, 610538), anti-CASPASE-3/cleaved CASPASE-3 (1:300; Wanleibio, WL02117), and anti-CASPASE-7/cleaved CASPASE-7 (1:500; Wanleibio, WL0181). Anti-glyceraldehyde-3-phosphate dehydrogenase (anti-GAPDH; 1:3,000; Millipore, MAB374, Billerica, MA, USA) was used as a loading control.

### Fluorescence-activated cell sorting

Four-month-old Tg (*mbp:nfsB-egfp*) (control group) and metronidazole treated Tg (*mbp:nfsB-egfp*) (Mtz treated group) zebrafish were anesthetized with 0.1% MS-222 and euthanized immediately. One brain from each group was isolated and washed twice in ice-cold PBS and dissociated into single-cell suspensions in 300 μL of ice-cold 0.25% trypsin - EDTA solution. GFP-positive (GFP^+^) cells were sorted using FACSAria III (Becton Dickinson, Franklin Lakes, NJ, USA) with a Coherent Innova 70 laser at 488 nm at 4°C. The same number of GFP^+^ cells was collected from the control group and the metronidazole treated group (2 × 10^5^ cells in each group). Then, total RNA from GFP^+^ cells from each group was extracted, and the expression of *IL-7R* was quantified using qRT-PCR as described above. Cross-intron primers were designed to detect whether the oligodendrocytes expressed *IL-7R*. The sequences were: forward 5′-TTTTCTGTCCTTTGGGCCTGT-3′; reverse 5′-CATTTTGCAGAGTGTTGCGA-3′. This experiment was repeated three times.

### Morpholino oligonucleotides, RNA synthesis, and microinjections

Morpholino oligonucleotides, either complementary to the translation start site of zebrafish *IL-7R* mRNA sequence (GenBank NM_001113507) or containing a 5-bp mismatch (control morpholinos), were designed and synthesized by Gene Tools, LLC (OR, USA). The sequences included *IL-7R* morpholino oligonucleotide (MO), 5′-ATCCACAAAAG TCCCGCCATCTCTC-3′ (the antisense start codons underlined); *IL-7R* mismatch oligonucleotide (MM), 5′-ATCgAgAAAAcTCCCcCgATCTCTC-3′ (the mismatch nucleotides underlined). Morpholino oligonucleotides were suspended in 1 × Danieau buffer at a concentration of 1 ng/nL and injected into one- to four-cell-stage wild-type embryos with 2 ng *IL-7R* MO or *IL-7R* MM [51, 52]. The injected embryos were cultured with sterilized 1×Holt buffer until 120 hpf.

*IL-7R* full-length coding sequences were obtained from zebrafish *IL-7R* mRNA sequence (GenBank NM_001113507) and subcloned into a pCS2 vector. mRNA was synthesized using SP6 mMessage mMachine kit (Thermo Fisher Scientific, Waltham, MA, USA). For the mRNA rescue injections, 10 pg of *IL-7R* mRNA was co-injected with 2 ng of *IL-7R* MO. Sterile water was used for the control experiment. The injected embryos were cultured with sterilized 1×Holt buffer until 120 hpf.

### Whole-mount *in situ* hybridization

Embryos or larvae were grown in 0.003% 1-phenyl-2-thiourea (PTU; Sigma) to block pigmentation and mediate visualization until 96 hpf. The whole-mount *in situ* hybridization was performed using a standard protocol [53, 54]. Three probes were used in this study. The oligodendrocyte lineage cells, including oligodendrocytes and Schwann cells, were labeled using an mRNA probe for *mbp* gene (GenBank AY860977). A *recombination activating gene 1* (*rag1*, GenBank NM_131389) mRNA probe was used as a marker to explore the development of the thymus; a *growth hormone1* (*gh1*, GenBank AJ937858) probe was used to label hormone-producing cells in the hypophysis [33]. The probes were added to Eppendorf tubes at a concentration of 2 ng/μL and hybridized overnight at 55°C. On the second day, the embryos were washed and incubated with an alkaline phosphatase-conjugated antibody (Roche Diagnostics) at a dilution of 1:1500. Nitro blue tetrazolium/5-bromo −4-chloro-3-indolyl-phosphate (NBT/BCIP; Roche) was used as an enzymatic substrate on the third day.

### Photography and image analysis

Immunohistochemical images were photographed with an FV 1000 confocal microscope (Olympus, Japan). Image J software (1.49 ×; NIH, http://rsb.info.nih.gov/ij/) was used to convert the fluorescent images of the GFP immunostaining to 8-bit grayscale prior to thresholding, and determining the thickness of myelin sheath and GFP positive number cells on each image. Images of embryos and whole-mount *in situ* hybridized larvae were photographed with a DP72 digital camera mounted on an SZX16 dissecting microscope (Olympus). All images were compiled in Adobe Photoshop 7.0 (Adobe, CA, USA) and resized. They were occasionally modified for contrast and brightness using the image/adjustments/contrast/brightness setting. All images within an experiment were manipulated similarly.

### Statistical analysis

All values are presented as the means ± SEM. GraphPad Prism Software (version 5.01 for Windows; GraphPad, San Diego, CA, USA) was used to perform the statistical analysis. To compare values from two or more independent groups, Student's *t* test or one-way ANOVA analysis of variance were performed. A *P value* less than 0.05 was considered statistically significant.

## SUPPLEMENTARY MATERIALS FIGURES


