# Supplementary material for: Down-regulation of interleukin 7 receptor (IL-7R) contributes to central nervous system demyelination
Source: Oncotarget. 2017 Mar 10;8(17):28395–407. doi: 10.18632/oncotarget.16081 (PMC5438658; doi:10.18632/oncotarget.16081)
Supplement: Supplementary file 1 [file oncotarget-08-28395-s001.pdf]

Down-regulation of interleukin 7 receptor (IL-7R) contributes to central nervous system demyelination

Supplementary Materials

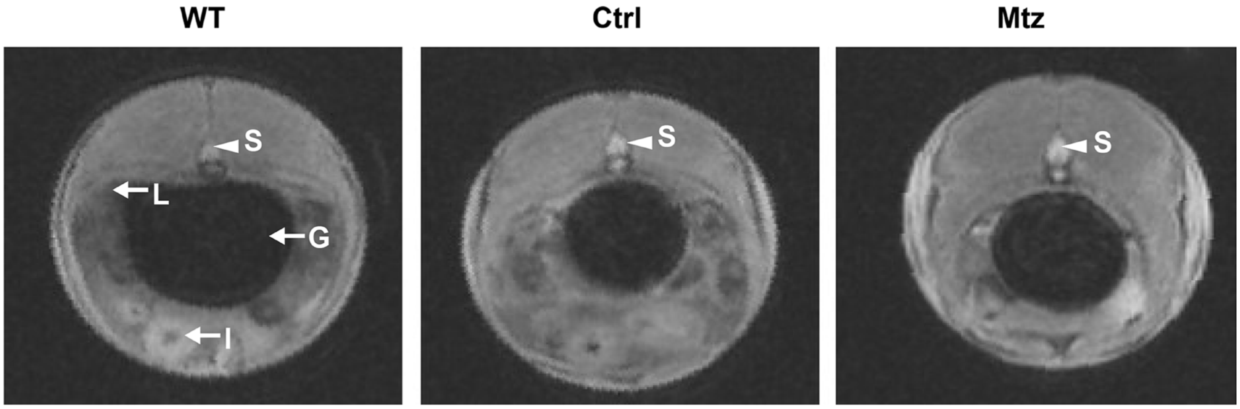

Supplementary Figure 1: Images of MRI scanning of cross-sections of the wild-type adult (WT), control Tg (*mbp:nfsB-egfp*) (Ctrl) and metronidazole treated Tg (*mbp:nfsB-egfp*) adults (Mtz). Abbreviations: S, spinal cord; G, gas bladder; L, liver; I, intestine.

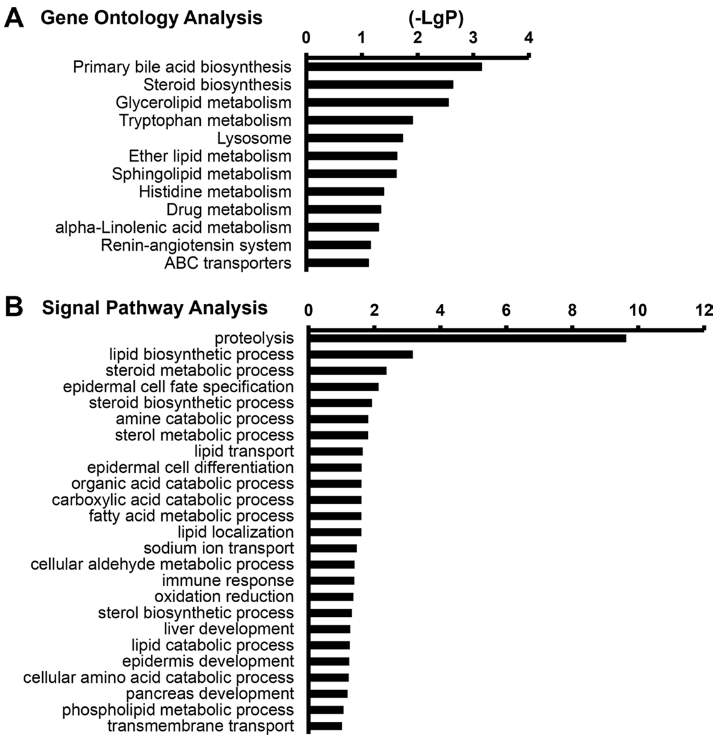

Supplementary Figure 2: Gene ontology (GO) and pathway analysis of the gene expression array. (**A**) The analysis of the relative changes in 12 GO clusters during demyelination. (**B**) The analysis of the relative changes in 25 pathways. -LgP: negative logarithm value of P value from differential gene expression by microarray.

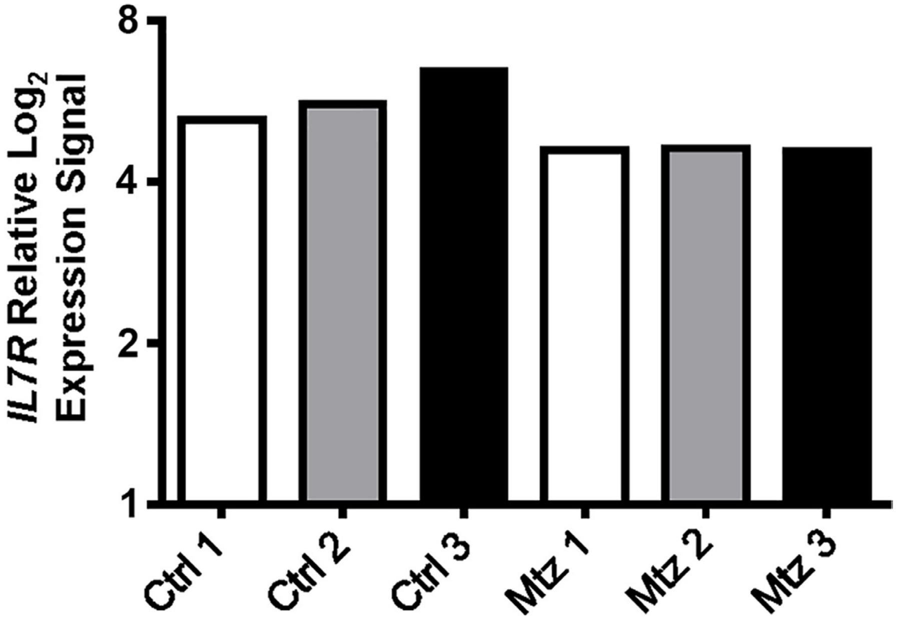

Supplementary Figure 3: The changes of *IL-7R* across the microarray in the three replicates. Abbreviation: Ctrl, control Tg (*mbp:nfsB-egfp*); Mtz, metronidazole treated Tg (*mbp:nfsB-egfp*).

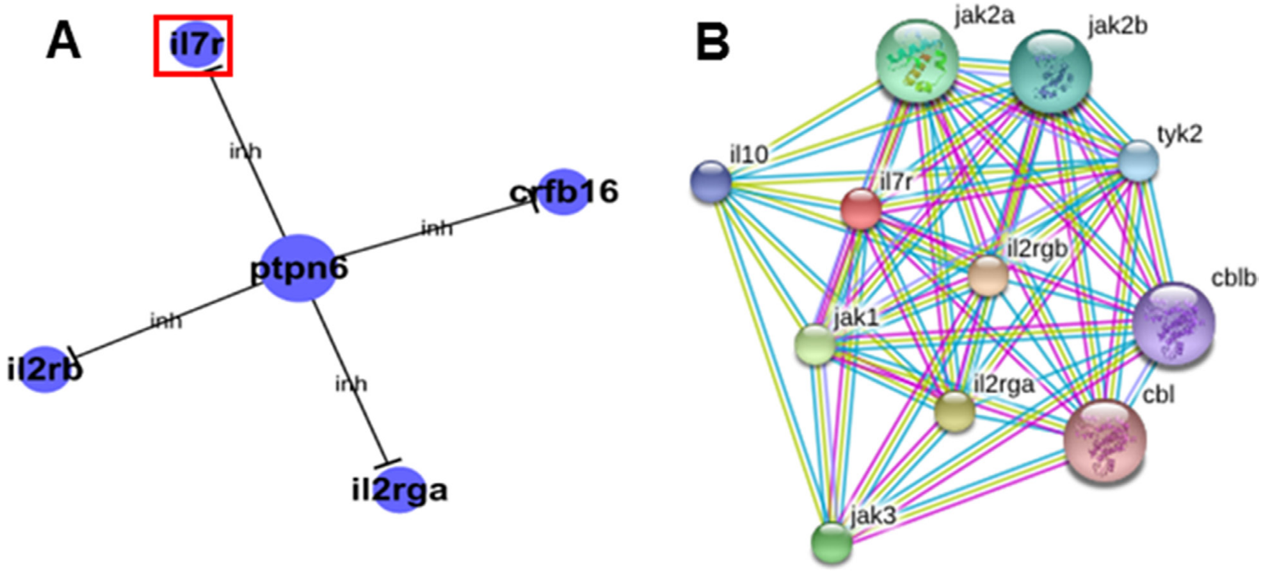

Supplementary Figure 4: Global signal transduction network on microarray. (**A**) *IL-7R* (red box) in global signal transduction network. (**B**) *IL-7R* String-db analysis. Small nodes: proteins with unknown 3D structure; large nodes: proteins with known or predicted 3D structure. Lines: significant transcriptional interactions; blue, known interactions from curated databases; magenta, known interactions experimentally determined; yellow, other interactions from textmining; lilac, other interactions from protein homology.
